# Factors Associated with Anxiety and Depression among Diabetes, Hypertension, and Heart Failure Patients at Dessie Referral Hospital, Northeast Ethiopia

**DOI:** 10.1155/2020/3609873

**Published:** 2020-05-16

**Authors:** Afework Edmealem, Caridad Sanchez Olis

**Affiliations:** Department of Nursing, School of Nursing and Midwifery, Wollo University, Dessie, Ethiopia

## Abstract

**Background:**

Anxiety and depression are common in patients with diabetes, hypertension, and heart failure. However, they are usually unrecognized and untreated especially in developing countries. Identifying factors associated with anxiety and depression is helpful for early screening and management.

**Objective:**

This study is aimed at assessing factors associated with anxiety and depression among diabetes, hypertension, and heart failure patients at Dessie Referral Hospital, Northeast Ethiopia.

**Methods:**

An institutional-based cross-sectional study was conducted in Dessie Referral Hospital from February 22, 2019 to April 6, 2019. A total of 404 diabetic, hypertension, and heart failure patients were included through systematic sampling technique. The data were collected by face-to-face interview. After data collection, the data were cleaned and presented with text, graphs, and tables. Multivariable binary logistic regression was deployed to identify factors at a *P* value of < 0.05.

**Result:**

A total of 384 patients participated with a 94.8% response rate. Among these, 32% and 5.73% of them had anxiety and depression, respectively. Patients who did not read and write develop anxiety 7.89 times more likely compared with those whose educational status is diploma and above (AOR: 7.89; 95% CI: 3.08-20.26; *P* = 0.001). Patients who took substances like chat, cigarette, shisha, hashish, and alcohol develop anxiety 2.56 times more likely compared with their counterparts (AOR: 2.56; 95% CI: 1.05–6.23; *P* = 0.038). Patients whose level of physical activity is inactive develop depression 24 times more likely than patients who did a health-enhancing physical activity. Patients who are widowed develop depression 5 times more likely compared with married patients. *Conclusion and Recommendations*. Low educational level, being single and widowed, substance use, poor perception towards prognosis of illness, and monthly income were factors associated with anxiety. On the other hand, being single and unable to do physical activity were statistically associated with depression. Patients with low educational level and monthly income should be screened and supported for anxiety. Health care providers should provide advice to patients about the importance of physical activity to prevent depression.

## 1. Introduction

Anxiety and depression are common mental illnesses. The total number of people living with anxiety and depression in the world is estimated to 322 million and 264 million, respectively [1]. In spite of this fact, anxiety and depression are common in diabetes, hypertension, and heart failure patients than the general population [2].

A systematic review and meta-analysis study reported that anxiety and depression were common among patients with chronic illness in both developed and developing countries [3]. Another systematic and meta-analysis conducted in Ethiopia indicates that depression is a common comorbid illness among patients with diabetes [4]. According to the national report of Australia Health, half of all Australians living with a chronic health condition experience depression or anxiety [5].

Anxiety and depression have a bidirectional relation with chronic illnesses [6, 7]. A review of anxiety disorders, hypertension, and cardiovascular risk indicated that hypertensive patients develop anxiety more likely [8]. Anxiety and depression causes chronic stimulation of sympathetic nervous system that results in insulin resistance and affects the function of the heart and blood vessels [6].

Having a chronic illness puts a person at greater risk of developing anxiety or depression. A study which is conducted in USA and China stated that both anxiety and depression are the major comorbidities in patients with chronic illnesses [9]. One recent study which is conducted in 15 nations reported that anxiety is highly prevalent in patients with chronic illness especially in diabetes patients [10]. However, anxiety and depression are remaining unrecognized and untreated.

Anxiety and depression have numerous negative health outcomes in patients with chronic illnesses. Medication nonadherence, rapid disease progression, and poor health outcomes were effects of untreated and unrecognized anxiety and depression [9]. Anxiety and depression are so overwhelming; they can interfere with a person's ability to function day to day and poor quality of life [11]. American Diabetes Association revealed that the mortality risk for diabetes patients is high in the presence of depression or anxiety or both [12].

There are different factors that affect the prevalence of anxiety and depression among patients with chronic illness. A systematic review and meta-analysis conducted in Ethiopia revealed that age, sex, and duration of disease were factors associated with depression [4]. Low educational level, body mass index, income, lack of social support, and residence of patients were the other factors associated with anxiety and depression [11, 13, 14].

Identifying factors associated with anxiety and depression is helpful for early screening and management. Nevertheless, factors associated with depression and anxiety in patients with diabetes, hypertension, and heart failure are not assessed adequately in developing countries including Ethiopia. Thus, this study was initiated to examine factors associated with anxiety and depression among patients with diabetes, hypertension, and heart failure in Dessie Referral Hospital, Northeast Ethiopia.

## 2. Methods

### 2.1. Study Area and Period

The study was conducted at Dessie Referral Hospital which is found in Dessie town. Dessie town, which is an administrative town of South Wollo Zone, is 401 km far from Addis Ababa in the northeast direction. Dessie Referral Hospital, which is one of the frontline hospitals in Ethiopia, serves more than 3.5 million people as a referral hospital. It has about 749 workers. From this, 548 are health professionals and 201 are administrative staffs serving in the hospital. Out of those health professionals, 332 are nurses and 61 of them are diploma nurses. The study was conducted from February 22, 2019 to April 6, 2019.

### 2.2. Study Design

An institutional-based cross-sectional study design was employed.

### 2.3. Source Population

All patients with diabetes, hypertension, and heart failure who are on follow-up in Dessie Referral Hospital were sources of population.

### 2.4. Study Population

All patients with diabetes, hypertension, and heart failure who are on follow-up in Dessie Referral Hospital during the data collection period were the study populations.

### 2.5. Inclusion and Exclusion Criteria

#### 2.5.1. Inclusion Criteria

- On follow-up patients who are diagnosed either with hypertension or diabetes or heart failure and who are 18 years and above were included

#### 2.5.2. Exclusion Criteria

- Patients unable to communicate were excluded

#### 2.5.3. Sample Size Determination

The sample size for the first objective was calculated by using the single-population proportion formula with 95% confidence level, 5% margin of error, and proportion of depression among patients with heart failure. Proportion, which is 51.1% %, was taken from a study conducted on depression among heart failure patients in three public hospitals of Northwest Ethiopia [15]. It was calculated as follows:

(1) $$\begin{array}{c} N=\frac{{\left(Z_{a/2}\right)}^{2}\left(P\right)\left(1-P\right)}{D^{2}}, \end{array}$$

where *N* is the sample size, *Z*_*a*/2_ = 1.96 (standardized normal distribution curve value for the 95% confidence interval), *P* = 0.8165 (proportion of depression among patients with heart failure) and *D* = 0.05 (degree of margin of error):

(2) $$\begin{array}{c} N=\frac{{\left(1.96\right)}^{2}\left(0.51\right)\left(0.49\right)}{{\left(0.05\right)}^{2}},\\ N=384.5,\\ N=\sim 385. \end{array}$$

Therefore, by adding a 5% nonresponse rate of 385, the total sample size was 404.

#### 2.5.4. Sampling Technique and Procedure

The study utilized a stratified random sampling technique. Initially, patients with a chronic illness were stratified into diabetes mellitus (DM), hypertension, and heart failure based on their diagnosis. After that, the total sample size was allocated for each stratum based on their proportion. Then, study participants were selected by systematic sampling in every *k*^th^ value which is 6 from each stratum. The *k* value was calculated from the proportion of sample in each stratum to the total population. The first patient was selected by simple random sampling from patients who are coming for follow-up during the data collection period. After that, data were collected in every 6^th^ patient from each stratum until the total sample size is achieved.

#### 2.5.5. Variables

Dependent variables were anxiety and depression, and independent variables were sociodemographic variables (sex, age, educational level, marital status, resident, occupation, monthly income, family size, weight, height, and BMI), disease characteristics (duration of disease since diagnosis, number of medication), perception towards prognosis of illness, substance and alcohol use (coffee and tea use, smoking, chat chewing, uses of hashish and shisha, and alcohol drinking), and physical activity.

### 2.6. Data Collection Tool and Procedures

#### 2.6.1. Data Collection Tool

The data were collected by using a structured questionnaire which is adapted from previous research. It has 3 parts. The first part asked about the sociodemographic status of the study participants. The second part measured the level of anxiety and depression with generalized anxiety disorder and patient health questionnaire, respectively. In this study, internal reliability for GAD-7 questionnaires and PHQ-2 questionnaires were 0.76 and 0.8, respectively. The third part focused on factors such as physical activity, substance use, alcohol use, and support from anyone. Physical activity was screened by the International Physical Activity Questionnaire (IPAQ-7) which is a standardized tool for measurement of physical activity for patients with chronic illnesses. All parts of the questionnaire were prepared in English version initially and translated into Amharic then back to English to check their consistency. Additionally, weight (in kilogram) and height (in meter) for nonpregnant and edematous patient were measured by data collectors during data collection.

#### 2.6.2. Data Collection Procedures

After preparing the questionnaire, 4 BSc nurses for data collection and 1 BSc nurse as a supervisor were recruited. A two-day training was given for each of them on the meaning of every items of the questionnaire and the techniques of data collection such as ways of greeting, ways of taking consent, ways of data quality monitoring during height and weight measurement, and ways of addressing ambiguous items. Data were collected by face-to-face interview after patients finish their visit.

Patients were interviewed with 7 questions to assess their level of physical activity. The total score of questions was categorized into three levels: inactive, those individuals who do not meet the criteria for minimally active or health-enhancing physical activity (HEPA); minimally active, 3 or more days of vigorous activity of at least 20 minutes per day or 5 or more days of moderate-intensity activity or walking of at least 30 minutes per day or 5 or more days of any combination of walking, moderate-intensity, or vigorous-intensity activities achieving a minimum of at least 600 metabolic equivalent (MET) (min/week); and health-enhancing physical activity, vigorous-intensity activity on at least 3 days achieving a minimum of at least 1500 metabolic equivalent (MET) (minutes/week) or 7 or more days of any combination of walking, moderate-intensity, or vigorous-intensity activities achieving a minimum of at least 3000 MET (minutes/week).

Height and weight were measured for nonpregnant and edematous patients during the data collection period by data collectors. To avoid a repeated interview for patients with repeated visit, data collectors asked and verified whether the patient is interviewed or not before data collection. The supervisor and principal investigator monitored closely the data collection process.

#### 2.6.3. Data Quality Assurance

The quality of data was assured by training the data collectors and supervisor, carefully designing the questionnaire, monitoring the data collection process, and checking the completeness of data during data collection time. In addition to these, all questionnaires were pretested on 10% of the sample size (40 respondents) at Hidar 11 Primary Hospital to address confusing items and to increase the quality of data. During the pretest, some respondents were confused on the duration of anxiety questions and physical activity questions. To address this confusing issue, data collectors tried to remind the duration in each section of the questionnaire at the time of data collection.

#### 2.6.4. Data Processing and Analysis Procedure

After data collection, completely collected data were entered in to EpiData version 3.1 and exported to Statistical Package and Service Product (SPSS) version 25 for analysis. During analysis, the study participants who scored 9 and above in the Generalized Anxiety Disorder item 7 (GAD-7) questionnaire were categorized as having anxiety, and study participants who scored 3 and above in the Patient Health Questionnaire item 2 (PHQ-2) questionnaire were categorized as positive for depression disorder. The results of the study was presented by using texts, tables, and figures, and the binary logistic regression model was enrolled by considering 95% confidence level and a *P* value of 0.05. Multivariable binary logistic regression was done by taking variables that have a *P* value of ≤ 0.2 from bivariable logistic regression to identify factors associated with anxiety and depression.

## 3. Result

### 3.1. Sociodemographic and Economic Characteristics

From a total of 405 respondents, 384 respondents participated with a 94.8% response rate. Among these, 179 (46.6%) were female, 152 (39.6%) were illiterate (unable to read and write), 241 (62.8%) were married, and 138 (35.9%) were farmers. The median age of respondents was 45 (IQR = 30), and 53 (13.8%) of the respondents were 65 and above years of age. From the total respondents, 151 (39.3%) of them lived in rural areas. Although 149 (38.8%) of the respondents had a family size of more than four, above one-fifth of the total respondents 84 (21.9%) did not get any support from others. BMI was calculated for 371 nonpregnant and edematous respondents, and among these, above one-fifth of them were overweight (BMI ≥ 25 kg/m^2^) (Table 1).

### 3.2. Disease Characteristics

From the total study participants, 39 (10.2%) had poor perception towards the prognosis of their illness. For 64 (16.7%) study participants, the duration of disease since diagnosis was above 6 years. From the total study participants, 26 (6.8%) took 5 and above drugs daily in the past one month (Table 2).

### 3.3. Substance and Alcohol Use

From the total study participants, majority 311 (81%) of them drank coffee and/or tea in the past one month. Only 4 (1%) respondents smoke cigarettes in the past month, and twenty-eight (7.3%) drank any type of alcohol in the past one month. From the sum score of alcohol drinking, smoking cigarette, chat chewing, and using shisha, and others, 32 (8.3%) of them took at least one substance in the past month (Table 3).

### 3.4. Physical Activity

**Figure 1:**
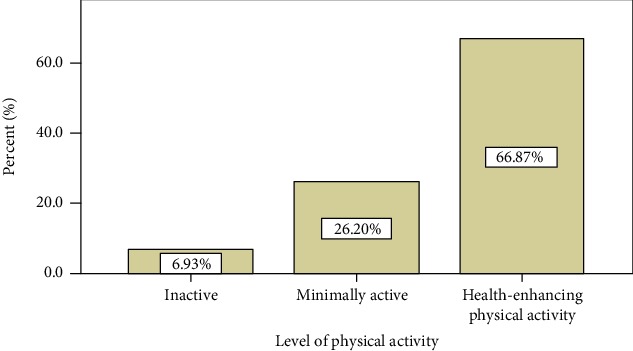
Level of physical activity among patients with diabetes, hypertension and heart failure at Dessie Referral Hospital, 2019.

Physical activity was assessed for only 332 study participants since others were excluded from the analysis during data cleaning because of incomplete data. Among these respondents, the level of physical activity for one-third 110 (33.1%) of them was inactive and minimally active (Figure 1).

### 3.5. Prevalence of Anxiety

**Figure 2:**
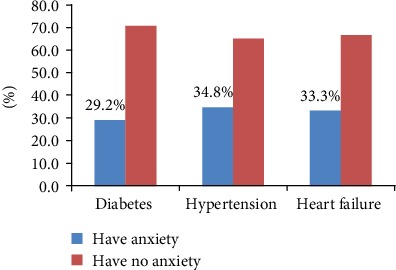
Prevalence of anxiety among patients with diabetes, hypertension and heart failure in Dessie Referral Hospital, Northeast Ethiopia, 2019.

From the total study participants, 123 (32%) had anxiety. Among the total hypertensive patients, 34.8% had anxiety. The prevalence of anxiety based on disease condition is presented in Figure 2.

### 3.6. Prevalence of Depression

**Figure 3:**
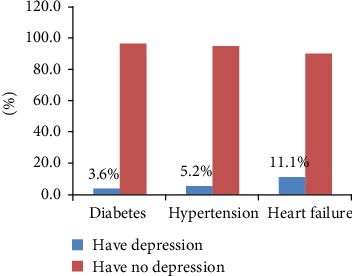
Prevalence of depression among patients with diabetes, hypertension, and heart failure at Dessie Referral Hospital, 2019.

In this study, among the total of 384 study participants, only 5.73% had depression. From the total heart failure patients, 11.1% had depression. The prevalence of depression based on the disease condition is presented in Figure 3.

### 3.7. Factors Associated with Anxiety

Variables which have an association with anxiety at *P* value ≤ 0.2 in bivariable logistic regression were educational status, marital status, occupation, residence, perception towards prognosis of illness, physical activity, body mass index, monthly income, supported by someone, and substance use (chat, cigarette, shisha, hashish, and alcohol). These were entered in multivariable logistic regression to identify factors associated with anxiety. However, in multivariable logistic regression, only educational level, marital status, monthly income, perception towards prognosis of illness, and substance use were associated with anxiety at *P* value < 0.05. According to the result, patients who did not read and write develop anxiety 7.89 times more likely compared with those whose educational status is diploma and above (AOR: 7.89; 95% CI: 3.08-20.26; *P* = 0.001). Patients who took substances like chat, cigarette, shisha, hashish, and alcohol develop anxiety 2.56 times more likely compared with their counterparts (AOR: 2.56; 95% CI: 1.05–6.23; *P* = 0.038) (Table 4).

### 3.8. Factors Associated with Depression

Variables which have an association with depression at *P* value ≤ 0.2 in bivariable logistic regression were sex, marital status, occupation, residence, perception towards prognosis of illness, physical activity, body mass index, number of drugs, and monthly income. These were entered in multivariable logistic regression to identify factors associated with depression. However, in multivariable logistic regression, only marital status and physical activity were associated with depression at *P* value < 0.05. According to the result, patients whose level of physical activity is inactive develop depression 24 times more likely than patients who did a health-enhancing physical activity (AOR: 24.03; 95% CI: 6.01–96.08; *P* = 0.001). Patients whose marital status is widowed develop depression 5 times more likely compared with patients who are married (AOR: 4.75; 95% CI: 1.25–18.05; *P* = 0.001) (Table 5).

## 4. Discussion

Anxiety and depression are common in patients with diabetes, hypertension, and heart failure. Despite this fact, there is lack of attention to screening and early treatment. Identifying risk factors is helpful for early screening and treatment. Thus, this study was carried out to assess factors associated with anxiety and depression among patients with diabetes, hypertension, and heart failure in Dessie Referral Hospital.

In this study, the prevalence of anxiety among patients with diabetes, hypertension, and heart failure was 32% (95% CI: 27.3%-36.7%). This finding is consistent with the finding of studies conducted in Korea (30%) [16] and Brazil (33.7%) [17]. This is lower than the finding of a study conducted in three Asian countries (Cambodia, Myanmar, and Vietnam) (17%) [18]. However, this finding was lower than studies conducted in Saudi Arabia (45%) [19]. The reason for this discrepancy could be the difference in study period, source population, and socioeconomic difference. In addition, the use of different tools could be the other reason for the discrepancy.

This study revealed that the prevalence of depression among patients with diabetes, hypertension, and heart failure was 5.73% (95% CI: 3.4%-8.3%). This finding is in line with the magnitude of depression among pregnant women in a study conducted at Malaysia (6.9%) [20]. However, this is lower than studies conducted in three public hospitals of Northwest Ethiopia (51%) [15], Addis Ababa (21%) [21], the pooled prevalence of depression among patients with diabetes in Ethiopia (39.73%) [22], Saudi Arabia (37.4%) [19], China (31.4%) [23], and the three Asian countries (39.1%) [18]. The possible justification for this discrepancy might be the difference in tool, source population, and socioeconomic difference. For example, in a study conducted in three public hospitals of Northwest Ethiopia, only patients with heart failure were included. In addition, a study conducted at Addis Ababa included asthmatic patients. In this study, patients with heart failure, hypertension, and diabetes were included.

In this study, anxiety had a significant association with low educational level. Patients who do not read and write develop anxiety 7.89 times more likely compared with those whose educational status is diploma and above (AOR: 7.89; 95% CI: 3.08-20.26; *P* = 0.001). The possible justification for this finding might be related with poor health perception and management in patients with low educational level. This finding is consistent with the report of study conducted in Pakistan among diabetes patients [14].

This study revealed that patients whose level of physical activity is inactive develop depression 24 times more likely than patients who did a health-enhancing physical activity (AOR: 24.03; 95% CI: 6.01–96.08; *P* = 0.001). The possible justification for this might be because of the stimulation of the hypothalamus and the production of endorphins during exercise. When endorphins are produced, they resemble the opiates and produce analgesia and sense of wellbeing. This is in line with the finding of a study conducted in Pakistan [24].

Marital status was statistically associated with depression. According to the finding, patients who were single reported depression 4 times more likely compared with married patients (AOR: 4.02; 95% CI: 1.05–15.41, *P* = 0.001). The possible justification for this might be the lack of social support for single patients. This finding was consistent with the finding of a study conducted in Addis Ababa [21], Saudi Arabia [19], and Spain [25].

The association between substance use and depression was not seen. This is consistent with the report of a study conducted in Addis Ababa [21]. However, the association between substance use and depression was reported in different studies [15, 26]. Unlike depression, substance use is statistically associated with anxiety. Patients who took substances like chat, cigarette, hashish, and alcohol develop anxiety 2.56 times more likely compared with their counterparts (AOR: 2.56; 95% CI: 1.05-6.23; *P* = 0.038). This might be because of withdrawal effect.

## 5. Limitation of Study

This study has limitations although it has different methodological strengths. However, having a cross-sectional study design is the limitation of this study. This study assessed anxiety and depression for only diabetes, hypertension, and heart failure patients. It cannot be generalized for other chronic illnesses.

## 6. Conclusion and Recommendations

Lower educational level, being single, higher monthly income, and poor perception to prognosis of illness were factors associated with anxiety among patients with diabetes, hypertension, and heart failure. On the other hand, being single and inactive to physical activity were factors associated with depression among patients with diabetes, hypertension, and heart failure. Any patient with diabetes, hypertension, and heart failure should be screened, recognized, and treated for anxiety and depression. Patients with low educational level and monthly income should be screened early for anxiety. Health care providers provide advice to patients with diabetes, hypertension, and heart failure about physical activity to prevent depression. Researchers should investigate more regarding the factors affecting depression and anxiety among patients with other chronic illnesses. Policy makers should develop a guideline for the screening and treatment of anxiety and depression.

## Figures and Tables

**Table 1:** Sociodemographic status of patients with diabetes, hypertension, and heart failure at Dessie Referral Hospital, 2019 (*N* = 384).

Variable	Category	Frequency	Percentage
Sex	Female	179	46.6
	Male	205	53.4

Age	18-24	52	13.6
	25-29	38	9.9
	30-34	30	7.8
	35-44	65	16.9
	45-64	146	38.0
	≥65	53	13.8

Educational level	Unable to read and write	152	39.6
	Able to read and write (informal school)	32	8.3
	Grade 1-8	53	13.8
	Grade 9-12	54	14.1
	Certificate	12	3.1
	Diploma and above	81	21.1

Marital status	Single	68	17.7
	Married	241	62.8
	Widow	53	13.8
	Divorced	22	5.7

Residence	Urban	233	60.7
	Rural	151	39.3

Occupation	Farmer	138	35.9
	Merchant	125	32.5
	Student	26	6.8
	Gov't or non gov't employee	84	21.9
	Others (retired, no permanent job)	11	2.9

Monthly income (ETB)	≤1000	113	29.4
	1001-2000	84	21.9
	2001-3500	96	25
	>3500	91	23.7

Family size	≤4	235	61.2
	>4	149	38.8

Any support	Yes	300	78.1
	No	84	21.9

BMI	Underweight	38	10.3
	Normal	255	68.7
	Overweight	78	21

Note: monthly income was categorized based on quartile range; family size was based on mean; BMI was based on WHO weight classification for Ethiopia.

**Table 2:** Disease characteristics and individual factors among patients with diabetes, hypertension, and heart failure at Dessie Referral Hospital, 2019 (*N* = 384).

Variable	Category	Frequency	Percent (%)
Perception towards prognosis of illness	Good	240	62.5%
	Fair	105	27.3%
	Poor	39	10.2%

Duration of disease after diagnosis	Below 3 years	163	42.4%
	3 to 6 years	157	40.9%
	Above 6 years	64	16.7%

Number of drugs taken daily	Below 5 drugs	358	93.2%
	5 and above drugs	26	6.8%

**Table 3:** Substance and alcohol use among patients with diabetes, hypertension, and heart failure at Dessie Referral Hospital, 2019 (*N* = 384).

Variable	Category	Frequency	Percent (%)
Coffee and/or tea	Yes	311	81
	No	73	19

Cigarette smoking	Yes	4	1
	No	380	99

Alcohol drinking	Yes	28	7.3
	No	356	92.7

Chat chewing	Yes	1	0.3
	No	383	99.7

Using shisha and other substances	Yes	0	0
	No	384	100

Sum of substance uses (alcohol drinking, chat chewing, cigarette smoking, shisha, etc.)	Yes	32	8.3
	No	352	91.7

**Table 4:** Bivariable and multivariable logistic regression output on the association between anxiety and factors, 2019 (*N* = 384).

Variable	Category	Anxiety		COR	AOR	*P* value
		No	Yes			
Educational level	Unable to read and write	86	66	1	1	0.001
	Able to read and write	23	9	0.51 (0.22-1.17)	7.91 (3.08-20.26)	
	Grade 1-8	41	12	0.38 (0.18-0.78)^∗^	3.81 (1.18-12.32)	
	Grade 9-12	36	18	0.65 (0.34-1.24)	1.18 (0.39-3.54)	
	Certificate	7	5	0.93 (0.28-3.06)	3.53 (1.30-9.57)	
	Diploma and above	68	13	0.24 (0.12-0.48)^∗^	3.83 (0.82-17.93)	

Marital status	Single	41	27	1.67 (0.95-2.93)	2.82 (1.35-5.91)	0.032
	Married	173	68	1	1	
	Widowed	33	20	1.54 (0.82-2.87)	0.80 (0.35-1.85)	
	Divorced	14	8	1.45 (0.58-3.62)	0.74 (0.23-2.40)	

Residence	Urban	173	60	0.48 (0.31-0.75)^∗^		
	Rural	88	63	1		

Occupation	Farmer	83	55	1		
	Merchant	85	40	0.71 (0.42-1.17)		
	Student	16	10	0.94 (0.39-2.23)		
	Employee	69	15	0.32 (0.17-0.63)^∗^		
	Others	8	3	0.56 (0.14-2.22)		

Monthly income	≤1000	65	48	1	1	0.001
	1001-2000	54	30	0.75 (0.42-1.34)	0.57 (0.28-1.17)	
	2001- 3500	76	20	0.35 (0.19-0.66)^∗^	0.25 (0.12-0.54)	
	>3500	66	25	0.51(0.28-0.92)	1.25 (0.53-2.93)	

Perception to prognosis of illness	Good	176	64	1	1	0.022
	Fair	63	42	1.83 (1.13-2.97)^∗^	2.28 (1.27-4.10)	
	Poor	22	17	2.12 (1.06-4.25)^∗^	1.41 (0.61-3.23)	

Substance use	Yes	17	14	1.99 (0.96-4.13)	2.56 (1.05-6.23)	0.038
	No	244	108	1	1	

Physical activity	Inactive	12	11	1.72 (0.72-4.09)		
	Minimally active	62	25	0.75 (0.44-1.30)		
	HEPA	145	77	1		

BMI	Undernutrition	23	25	1.30 (0.64-2.62)		
	Normal	170	85	1		
	Overweight	59	19	0.64 (0.36-1.14)		

Notes: Hosmer and Lemeshow test = 0.954; ^∗^Significant variables at *P* value < 0.05 in bivariable logistic regression.

**Table 5:** Bivariable and multivariable logistic regression output on the association between depression and factors, 2019 (*N* = 384).

Variable	Category	Depression		COR	AOR	*P* value
		No	Yes			
Sex	Female	165	14	2.08 (0.85-5.10)		
	Male	197	8	1		

Marital status	Single	62	6	3.23 (1.04-9.97)^∗^	4.02 (1.05-15.41)	
	Married	234	7	1	1	0.001
	Widowed	45	8	5.94 (2.05-17.21)^∗^	4.75 (1.25-18.05)	
	Divorced	21	1	1.59 (0.18-13.56)		

Residence	Urban	225	8	0.34 (0.14-0.85)		
	Rural	137	14	1		

Occupation	Farmer	128	10	1		
	Merchant	116	9	0.99 (0.39-2.52)		
	Student	24	2	1.06 (0.22-5.17)		
	Employee	84	0			
	Others (retired, no job)	10	1	1.28 (0.14-11.03)^∗^		

Monthly income	≤1000	101	12	1		
	1001-2000	78	6	0.64(0.23-1.80)		
	2001–3500	94	2	0.18(0.03-0.82)^∗^		
	>3500	89	2	0.18(0.04-0.87)^∗^		

Perception to prognosis of illness	Good	232	8	1		
	Fair	96	9	2.71 (1.01-7.25)^∗^		
	Poor	34	5	4.26 (1.31-13.79)^∗^		

Substance use	Yes	331	21	0.5 (0.06-3.90)		
	No	31	1	1		

Physical activity	Inactive	16	7	18.98 (5.41-66.6)^∗^	2.13 (0.58-7.83)	
	Minimally active	81	6	3.21 (0.95-10.82)^∗^	24.03 (6.01-96.0)	
	HEPA	217	5	1	1	0.001

BMI	Undernutrition	33	5	2.82 (0.94-8.42)		
	Normal	242	13	1		
	Overweight	75	3	0.74 (0.20-2.68)		

Number of drugs	<5 drugs	339	19	1		
	≥5 drugs	23	3	2.32 (0.64-8.44)		

Notes: Hosmer and Lemeshow test = 0.709; ^∗^Significant variables at *P* value < 0.05 in bivariable logistic regression.

## Data Availability

The data used to support the findings of this study are available from the corresponding author upon request.
